# Columbia Dental Club of Chicago

**Published:** 1893-07

**Authors:** 


### COLUMBIA DENTAL CLUB OF CHICAGO.

THIS club has been organized for the entertainment of visiting dentists from all parts of the world and to serve as head-quarters for the World's Columbian Dental Congress.

The dentists of Chicago have solved one of the greatest difficulties connected with the Congress, that of having a place where visiting dentists can meet. The stopping-places will be necessarily widely scattered, and it seemed at one time as though the social life of the Congress would be limited to the meetings. This admirable arrangement entirely overcomes this difficulty.

A Bureau of Information has also been established in connection with it, through which convenient rooms may be secured at rates to suit. Address Dr. R. C. Brophy, Manager of the Bureau, 300 Michigan Avenue, Chicago. Those who wish can have their mail directed to this number.

The efforts of the profession in Chicago to make it comfortable for visitors deserve the highest praise, and we have no doubt will be gratefully appreciated by all dentists in attendance.

